# FE-SEM/EDX Based Zinc Mobilization Analysis of *Burkholderia cepacia* and *Pantoea rodasii* and Their Functional Annotation in Crop Productivity, Soil Quality, and Zinc Biofortification of Paddy

**DOI:** 10.3389/fmicb.2022.852192

**Published:** 2022-05-06

**Authors:** Viabhav Kumar Upadhayay, Ajay Veer Singh, Amir Khan, Jyoti Singh, Navneet Pareek, Alok Raghav

**Affiliations:** ^1^Department of Microbiology, College of Basic Sciences and Humanities, G. B. Pant University of Agriculture and Technology, Pantnagar, India; ^2^Department of Soil Science, College of Agriculture, G. B. Pant University of Agriculture and Technology, Pantnagar, India; ^3^Multidisciplinary Research Unit, Department of Health Research, Ministry of Health and Family Welfare, Ganesh Shankar Vidyarthi Memorial Medical College, Kanpur, India

**Keywords:** FE-SEM/EDX, biofortification, zinc solubilizing bacteria, zinc, rice

## Abstract

The experimental study was contrived to characterize two zinc-solubilizing bacteria (ZSB), namely BMRR126 and BMAR64, and their role in zinc (Zn) biofortification of rice. These bacteria solubilized Zn profoundly, determined qualitatively by halo-zone formation on a solid medium and quantitatively in a liquid broth by AAS and SEM-EDX. The lowering of pH and contact angle assessment of the liquid broth unveiled the establishment of the acidic conditions in a medium suitable for Zn solubilization. The characterization of both isolates on the basis of 16S rRNA gene analysis was identified as *Burkholderia cepacia* and *Pantoea rodasii*, respectively. These strains were also found to have some plant probiotic traits namely phosphate solubilization, production of siderophore, indole acetic acid (IAA), exopolysaccharide (EPS), and ammonia. The field experiments were performed at two diverse locations and under all treatments; the simultaneous use of BMRR126 and BMAR64 with zinc oxide (ZnO) resulted in the highest growth and productivity of the paddy crop. The utmost Zn achievement in the grain was estimated in a treatment (T9) (25.07 mg/kg) containing a consortium of BMRR126 and BMAR64 along with ZnO for the *Terai region*. The treatment containing single ZSB bioinoculant BMRR126 (T7) showed an elevated Zn amount in the rice grain (33.25 mg/kg) for the *Katchar region*. The soil parameters (pH, EC, organic carbon, NPK, available Zn, and dehydrogenase activity) were also positively influenced under all bacterial treatments compared to the uninoculated control. Our study clearly accentuates the need for Zn solubilizing bacteria (ZSB) to provide the benefits of Zn-biofortification in different regions.

## Introduction

Agriculture is the topmost significant sector in India, besides industrial and technological developments, which contributes to a prolific role in 17.5% of the Nation’s GDP (Sambasivam et al., 2020). Rice is an important edible staple crop that fulfills the nutritional requirements of an ever-growing population of the world (Val-Torregrosa et al., 2021). The addition of high-yielding rice varieties and the application of agrochemicals (fertilizers and pesticides) resulted in a significant augmentation in the rice yield (Eliazer Nelson et al., 2019; Yu et al., 2020). However, it can be predicted that rice production will be affected adversely in the near future due to some issues, such as the shrinking of rice acreage, shortage of water for proper irrigation, and higher cultivation costs. Furthermore, micronutrient dearth is the most important limiting factor for global rice productivity (Senthilkumar et al., 2021). Micronutrients, especially Zn, are required in very minute quantities and show a crucial job in the well-being of humans and plants (Sharma et al., 2013; Natasha et al., 2022). Current studies focus on the modern agricultural production of Zn-fortified food crops to curtail the negative effects of Zn-associated malnutrition among a deprived section of the global population (Kiran et al., 2022; Naeem et al., 2022). Although soils contain a huge portion of the insoluble Zn which is not accessible by plants (Moreno-Lora and Delgado, 2020), and thereby affects the amount of Zn in grains (Liu et al., 2019).

An ample range of soil factors, typically total Zn level, elevated pH, and the maximum content of calcite, organic matter, Ca, Mg, Na, phosphate, and bicarbonate also influence the Zn availability in plants (Alloway, 2009). The inadequacy of Zn exhibits various symptoms that typically become apparent within 2 to 3 weeks after seedling transplantation of rice. Symptoms may include the incidence of “brown blotches: and “streaks,” which may coalesce to obscure older leaves completely, stunted growth, delay in maturity, and decline in yield (Wissuwa et al., 2006; Singh and Prasad, 2014). If we consume the rice grown on Zn scarce soil, it can lead to Zn malnutrition in humans (Rehman et al., 2012). To circumvent the issue of Zn deficiency, few methods are popular. The foremost agronomic method entails the usage of Zn fertilizers as a soil application (Liu et al., 2020) or as a foliar spray (Poblaciones and Rengel, 2017; Cakmak and Kutman, 2018). But the utilization of this method depicts environmental and economical pressure (Wissuwa et al., 2006), and Zn fertilizers convert to insoluble forms within 7 days of application (Rattan and Shukla, 1991; Hussain et al., 2018). Other methods involve three important approaches that include breeding (Swamy et al., 2016), genetic engineering (Mhatre et al., 2011; Das et al., 2020), and transgenics (Malik and Maqbool, 2020). However, all these approaches are costlier, slower, and laborious (Upadhayay et al., 2019). Studies on the functional aspects of zinc solubilizing bacteria (ZSB) can better replace these approaches (Kamran et al., 2017; Mumtaz et al., 2017; Upadhayay et al., 2021, 2022).

As a part of both the rhizo-microbiome and the phyto-microbiome, ZSB exhibits a benevolent role in maintaining plant health. The contemporary understanding of ZSB inoculants and their significance in Zn-biofortification has been imperceptibly explicated (Sharma et al., 2012; Costerousse et al., 2017; Kamran et al., 2017; Mumtaz et al., 2017; Hussain et al., 2018; Bhatt and Maheshwari, 2020; Kushwaha et al., 2020, 2021; Upadhayay et al., 2021, 2022; Prathap et al., 2022). Few studies displaying an interaction between plants and ZSB inoculants for better plant growth provided expectations to resolve the issue of Zn malnutrition in an eco-friendly manner (Ramesh et al., 2014; Kamran et al., 2017; Hussain et al., 2018; Bhatt and Maheshwari, 2020; Kushwaha et al., 2020; Bashir et al., 2021; Rezaeiniko et al., 2022; Upadhayay et al., 2022). The usage of ZSB provides an economically feasible tactic for the Zn biofortification of food crops (Upadhayay et al., 2022). The ZSB solubilizes the complex form of Zn found in the soil into a simpler form, thus giving plants access to the proper level of Zn (Saravanan et al., 2004; Kushwaha et al., 2020). Wide arrays of mechanisms involved for Zn solubilization include organic acid secretion (Costerousse et al., 2017), production of chelating agents (Singh and Prasanna, 2020), protons (Kushwaha et al., 2020), and oxidoreductive systems on cell membranes. Organic acids produced by ZSB show a downward trend in pH in the nearby soil, thus creating a suitable environment for Zn solubilization (Alexander, 1997; Wu et al., 2006; Kamran et al., 2017; Upadhayay et al., 2018, 2022). Other mechanisms include the secretion of iron-chelating agents, “siderophores,” which are believed to play a key role in solubilizing micronutrients, such as Fe and Zn (Upadhayay et al., 2018, 2022).

Few studies also described the plant probiotic traits of ZSB, such as “phosphate solubilization,” production of “siderophore,” “phytohormones,” “exopolysaccharides,” “HCN,” and “ammonia” (Kamran et al., 2017; Mumtaz et al., 2017; Bhatt and Maheshwari, 2020). These features make ZSB an excellent plant probiotic strain to facilitate crop growth besides providing the benefit of biofortification. Many ZSB strains namely *Acinetobacter*, *Burkholderia* (Vaid et al., 2014), *Bacillus* (Zeb et al., 2018), *Enterobacter*, and *Sphingomonas* (Wang et al., 2014) improved the Zn level in various regions of rice plant. The consortium of ZSB, which consists of the three strains, *Agrobacterium* sp., *A*. *lipoferum*, and *Pseudomonas* sp., showed an improvement in rice productivity in a field-based study (Tariq et al., 2007). Other prospective ZSB strains augmented Zn in the edible portion of soybean (Sharma et al., 2012), wheat (Rana et al., 2012), and maize (Mumtaz et al., 2017).

Considering the above facts, the current study was conducted to reveal the zinc oxide (ZnO) solubilization capability of two bacterial strains isolated from the rhizosphere of barnyard millet. Bacterial strains were then tested for their functional plant probiotic traits, followed by field trials conducted at two consecutive sites, especially in the “*Terai*” and “*Katchar*” regions of northern India, to illustrate the impact of the ZSB and their consortium (with and without ZnO supplementation) on growth-yield related parameters of rice crops and soil quality. Furthermore, the main objective of decoding the benefit of Zn-biofortification through estimation of Zn content in grains under the influence of ZSB was achieved.

## Materials and Methods

### Soil Collection and Microorganisms

The samples of rhizospheric soil were collected from “Barnyard millet” (underutilized crop) growing at high altitudes of the Northern Himalayan regions, such as Ranichauri (30.3111°N, 78.4097°E) and Almora (29.8150°N, 79.2902°E). The carefully taken samples were tightly packed in sterilized sample collection bags and immediately brought to the microbiology laboratory for further studies. About 1 g of soil sample was serially diluted (up to 10^6^) in the saline solution. About 100 μl aliquot of the diluted samples was homogeneously spread on NA (nutrient agar) medium plates. The bacterial colonies with different morphological properties were then selected after the plates had been properly incubated for 24 h at 28 ± 2°C. Afterward, the cultures of bacteria were preserved at −20°C (in glycerol stock) and in slants at 4°C for regular use.

### Zinc Solubilization Bioassay

The preliminary qualitative screening for the selection of potential Zn mobilizing rhizobacteria was executed by adapting the protocol of Ramesh et al. (2014). In brief, Tris minimal medium agar plates containing 0.1% of complex zinc source i.e., “ZnO” were spot inoculated with freshly grown isolates on NA plates. The inoculated Petri plates, after sealing with the parafilm, were kept for incubation for 1 week (at 28 ± 2°C). After incubation, the appearance of the halo zone around the bacterial colonies reflected a “detecting point” for deciphering the eventuality of zinc solubilization, and the halo zone was measured in centimeters. The formation of a halo zone around bacteria is attributed due to the secretion of organic acids that lower the pH of the nearby milieu and solubilize the complex form of zinc, and this phenomenon appears in the form of a “halo zone.”

Furthermore, the Zn solubilization efficiency (ZnSE) of the ZSB strains was calculated in percentage (%) by using the following formula:


ZnSE=diameterofsolubilizationhalo/diameterof thecolony×100

The selected ZSB were also determined for quantitative Zn mobilization in Tris-minimal liquid broth medium (with ZnO as the insoluble Zn source) by adapting the procedure of Fasim et al. (2002). In this process, 50 ml of ZnO-supplemented basal liquid medium was inoculated with 500 μl aliquots of overnight grown ZSB inoculums and kept for incubation by providing constant temperature (28 ± 2°C) for 10 days in an orbital shaker at 130 rpm while the ZnO-supplemented media without bacterial inoculation was maintained as a non-inoculated control. After proper incubation, the inoculated and uninoculated liquid broth was centrifuged at 6,100 × *g* (10 min) followed by filtration through Whatman filter paper (No. 42). The soluble Zn in the filtered suspension was evaluated by atomic absorption spectrophotometer (AAS). The pH of the suspension was also assessed by a pH meter for predicting the organic acid production in the liquid medium pertaining to Zn solubilization by following the experimental procedure of Mumtaz et al. (2017). The contact angle of the supernatant acquired from the liquid medium was also measured for observing the impact of ZSB on the inoculated broth. Contact angles were measured from the two opposite sides, which are represented as the left contact angle and right contact angle, respectively, and repeated three times.

### Field Emission-Scanning Electron Microscopy/Energy Dispersive X-ray Analysis

The Zn-solubilizing behavior was also determined by Field Emission-Scanning Electron Microscope (FE-SEM) and energy dispersive X-ray (EDX). For this process, 50 ml of ZnO-amended Tris mineral-based medium was inoculated with 500 μl aliquots of fresh test bacterial culture and kept incubated at 28 ± 2°C in an orbital shaker (at 130 rpm). The ZnO-supplemented media deprived of bacterial inoculation was kept as an uninoculated control. On the 10th day after the incubation, the medium suspension of the flask was centrifuged at 10,000 rpm for 10 min. The medium suspension of the flask was centrifuged at 10,000 rpm for 10 min. After discarding the supernatant, the resulting residues were washed three times with double distilled water (DW) and dried. The dried samples were mounted on a carbon tape attached to a brass holder as per the method of Delvasto et al. (2009). The assembled samples were then sputter-coated with a carbon gold double layer. SEM and EDX-based evaluation for elements was accomplished using a JSM-7100F field emission SEM in conjunction with EDX.

### Characterization of Bacterial Isolates

Various characteristics, namely colony morphology, Gram staining, cell morphology, and endospore formation, were determined by standard methods. The biochemical behaviors of the selected ZSB strains were identified using the Bergey’s Manual of Determinative Bacteriology. The genomic DNA of both bacterial strains was isolated as per the method described by Bazzicalupo and Fani (1995). The 16S rRNA gene for each bacterial isolate was amplified in 100 μl reaction mixture containing 1 μl template DNA, 4 μl dNTPs (2.5 mM each), 10 μl 10X Taq DNA polymerase assay buffer, 1 μl Taq DNA polymerase enzyme (3 U/μl), water and primer (400 ng of each forward primer: 5′–CAGGCCTAACACATGCAAGTC–3′ and reverse primer: 5′– GGCGGATGTGTACAAGGC – 3′). Initial denaturation was provided at 95°C for 5 min, followed by 35 cycles of 94°C for 30 s, 50°C for 30 s, 72°C for 1.5 min, and final extension at 72°C for 7 min. The ∼1.5 kb, 16S-rDNA fragment was amplified using a thermal cycler “PTC-200 thermal cycler” (M.J. Research) and sequenced through Chromus Biotech (Bangalore, India). The identification of ZSB isolates was performed based on the 16S rRNA gene sequence homology by the MEGA6 software using a neighbor-joining method with 1,000 bootstrap replicates (Tamura et al., 2011).

### *In vitro* Screening of PGP Traits

#### Phosphate Solubilization

Quantitative analysis for determining phosphate solubilization potential of both isolates was assessed by the method of Nautiyal (1999). The fresh cultures (1 ml) of test isolates were inoculated into 50 ml of NBRIP broth. After incubation at 28 ± 2°C (120 rpm) for up to 7 days, 5 ml of the suspension was drawn out and filtered through Whatman No. 1 filter paper, which was then centrifuged at 10,000 rpm (for 20 min). After centrifugation was complete, 1 ml of cell-free suspension was mixed with 2.5 ml of Barton’s reagent and the volume was made up to 50 ml by the addition of distilled water. After the development of a yellow color (10 min incubation), the OD of the suspension was recorded by a spectrophotometer and the total amount of dissolved P was determined from the standard curve.

#### Indole-3-Acetic Acid Synthesis

The synthesis of plant hormones, such as indole-3-acetic acid (IAA) by both strains was analyzed using Salkowski’s reagent as per the protocol of Patten and Glick (2002). The bacterial cultures, grown in Luria broth (supplemented with 50 μg/ml of tryptophan) at 28 ± 2°C for 4 days, were centrifuged at 5,500 rpm for 10 min (at 4°C). About 2 ml aliquots of the supernatant were collected into fresh test tubes, followed by the addition of orthophosphoric acid (2 drops) and Salkowski’s reagent (4 ml). The appearance of the pink color indicated the bacterial production of IAA in the broth medium and the OD was taken at 540 nm. The production of IAA in the broth medium was evaluated by comparison to the standard curve of IAA.

#### Siderophore Production

Production of siderophore (iron chelating agent) was estimated on Chrome-Azurol S (CAS) medium as presented by Schwyn and Neilands (1987). For performing this, the CAS agar plates were spot-inoculated with fresh bacterial culture. After 4 days of incubation at 28 ± 2°C, siderophore production was confirmed based on the measurement of the yellow-orange halo zone that appeared around the bacterial colony.

#### Production of Exopolysaccharide and Ammonia

To assess the EPS production ability of selected bacterial isolates, the 500 μl aliquot of a freshly grown bacterial suspension grown in the nutrient broth was inoculated into 50 ml of special medium for EPS production as suggested by Siddikee et al. (2011). After incubation at 28 ± 2°C for 5 days with shaking at 150 rpm, the broth was centrifuged at 10,000 × *g* (10 min). The resultant supernatant was collected in a fresh and sterile test tube, and after adding three-fold chilled absolute ethanol, the tubes were kept at 4°C overnight. The formation of the precipitation in the suspension indicated a positive result for EPS production. Once this step was completed, centrifugation was performed at 7,000 × *g* (20 min), the supernatant was discarded, and the resulting pellet was collected. The pellet was dried at 60°C for the complete disappearance of the alcohol and the dry weight of the dried residues was calculated in mg/ml. For the production of ammonia by bacterial isolates, the method of Cappuccino and Sherman (1992) was used. Vials containing peptone water (5 ml/vial) were inoculated with freshly grown bacterial cultures and incubated at 28 ± 2°C for 48 h. At the end of the incubation period, Nessler’s reagent (0.5 ml) was added to each vial and kept at room temperature for at least 5 min, and the positive result was assessed by the development of a brown to yellow color.

### Bacterial Culture Conditions and Consortium Preparation

The characterized ZSB isolates (BMRR126 and BMAR64) were cultured on Luria-Bertani (LB) agar plates at 28 ± 2°C for 24 h. Bacterial isolates were subcultured onto a fresh medium every 2 weeks. For the preparation of liquid cultures, the fresh colony of the bacterial culture was transferred to an LB broth medium and kept for incubation at 28 ± 2°C (for 48 h) with shaking at 200 rpm. Afterward, the broth with visible bacterial growth was spun in a centrifuge for 15 min (at 6000 × g) and the resulting bacterial pellet was suspended in sterile distilled water to achieve a concentration of 10^9^ CFU/ml. This concentration of the bacterial strains was used for various *in vitro* assays. Before the preparation of the bacterial consortium, the biocompatibility of both strains was also assessed according to the method of Roshani et al. (2020). A freshly grown colony on LB agar plate from each compatible culture was inoculated into a test tube containing 10 ml of nutrient broth (NB) medium and kept at proper incubation for 24 h (with constant shaking at 120 rpm) at 28 ± 2°C. The absorbance of bacterial grown suspension was analyzed at the optimum wavelength (600 nm). Afterward, the equal culture volume {A_600_-0.6} from both compatible bacterial cultures was dispensed to 100 ml of NB and mixed properly to build a bacterial consortium (Prasad and Babu, 2017).

### *In situ* Field Study

#### Site Description and Experimental Design

The experimental study was executed at two subsequent locations in the “*Terai*” region - Breeder Seed Production Center (BPSC), GBPUA&T, Pantnagar (U.S.Nagar), Uttarakhand, India (29.0308° N, 79.4651° E) and the “*Katchar*” region - Village: Kariyamai (Budaun), Uttar Pradesh, India (28.2523° N, 78.7490° E) from July 2018 to October 2018. The soil characteristics of the experimental area in both regions are provided in Supplementary Table 3. The research trials were set up in a randomized block design (RBD) with three replications. The trial included nine treatments mentioned in Table 1.

**TABLE 1:** Treatments used for field trial.

Labels	Treatments Description	Labels	Treatments Description
T1	Control	T6	Consortium***
T2	ZnSO_4_*	T7	BMRR126 + ZnO
T3	ZnO**	T8	BMAR64 + ZnO
T4	BMRR126	T9	Consortium*** + ZnO
T5	BMAR64		

*ZnSO_4_ as Zn supplement @25 kg/hectare; **ZnO as Zn supplement @60 kg/hectare; ***Consortium contains zinc solubilizing bacterial (ZSB) strains, BMRR126 and BMAR64.

#### Raising of Nursery

The preparation of nursery beds was carried out in both sites (*Terai* and *Katchar*) with the proper inclusion of drainage channels along the bed to drain the excess water. The seeds of the rice variety “*Pusa Basmati-1*,” which were procured from the Breeder Seed Production Center, Pantnagar (Uttarakhand), India, were soaked for 48 h and kept in a moist gunny cloth for 48 h. The seeds were broadcasted uniformly on the previously established nursery beds. Adequate moisture levels were maintained by irrigation, but flooding was avoided.

#### Trial Establishment

Field preparation at both sites (*Terai* and *Katchar*) was commenced a week before transplanting. Some essential procedures as suggested by agricultural experts were followed to prepare the farmland for field trial. The main field was ploughed with tractor-drawn disc plough followed to obtain a good tilth, and the bunds (up to 15 cm in height) were made in dry conditions after being compacted. Each plot was 4 m long and 3 m wide, giving a total area of 12 m^2^. Each plot was manually leveled and the soil was saturated prior to transplanting. The rice seedlings of 21 days old were uprooted from a nursery and thoroughly washed with water, and precautions were taken to have a lesser degree of damage to the plant roots. Uprooted seedlings were taken from the nursery to the transplanted site, followed by immersion of the roots of seedlings in the LB liquid broth of test bacterial suspensions (individual ZSB strain and consortium) with an adequate bacterial population density of approximately 10^6^ to 10^7^ cfu/ml as per the method of Tariq et al. (2007), Sharma et al. (2014). Inoculated seedlings (three seedlings per hill) were transplanted manually by maintaining a planting geometry of 15 cm × 20 cm. The recommended dose of fertilizers was applied before transplantation. The recommended doses of ZnSO_4_ (@25 kg/hectare) and ZnO (@60 kg/hectare) were broadcasted into the plots according to the treatment. Irrigation, fertilization, weed management, and plant protection were performed according to standard procedures. The trials at both sites were maintained until crop harvesting. The detail of the trial establishment is given in Table 2.

**TABLE 2:** Details of trial establishment.

Particular	Details
Design	Randomized Block Design (RBD)
Crop	Rice (*Oryza sativa*)
Variety	*Pusa Basmati* 1
Number of replications	3
Number of treatment combinations	9
Total number of plots	27
Gross plot size	12 m^2^
Net plot size	6.6 m^ 2^
Spacing	15 × 20 cm

#### Biometric Observation and Yield Assessment

At the time of harvest, biometric observations, such as the height of the plant, number of tillers (per hill), and dry matter accumulation were recorded. The four main yield components, such as the panicle length, the number of panicles per hill, the number of grains per panicle, and the 1,000 grain weight were also determined. The crop was harvested for decoding yield assessment, such as biological yield, grain yield, straw yield, and harvest index, when the plants attained physiological maturity. To circumvent the border effect, border rows were first harvested before net plots were harvested. After 72 h of sun drying in the field, the produce of each plot was tied into bundles (plot-wise), weighed in kg per plot with a spring balance, and then converted into q ha^–1^ for assessing the biological yield. After threshing the bundles, the grain and straw yield was recorded in kilograms per plot and then expressed in q ha^–1^. The harvest index of each plot was calculated by the following formula:


Harvestindex=Economicyield/Biologicalyield×100

### Zinc Estimation in Grain

Grain samples harvested after 120 days after transplanting (DAT) of each treatment were further analyzed for Zn content according to the study by Estefan et al. (2013). In brief, 0.1 g of paddy grain samples were weighed and transferred into the flask. About 10 ml of a triacid mixture consisting of nitric acid: sulfuric acid: perchloric acid (10: 1: 4 v/v/v) were added to each flask. The flasks were then placed on a hot plate (95°C) for complete digestion. Later, 5 ml of 6N HCl was added after completion of digestion and the volume was made up to 50 ml by adding distilled water. The digested solution was filtered through a filter paper (Whatman No. 1 filter paper) and transferred to fresh storage vials. The filtered digested samples were then used to analyze the Zn content in the samples by an atomic absorption spectrophotometer (AAS).

### Soil Parameters

At the time of harvest, the soil samples were collected from each plot to determine different parameters, such as pH, EC, available Zn, organic carbon (%), and available NPK. The pH and EC were determined by following the procedure of Bower and Wilcox (1965), Jackson (1967), respectively. The estimation of the available Zn in the soil of each plot was performed using the DTPA extraction method (Lindsay and Norvell, 1978). While the organic carbon content was estimated by adapting the standard protocol (rapid titration method) of Walkley and Black (1934), the availability of macronutrients, such as N, P, and K, was assessed using the procedures of Hanway and Heidel (1952), Olsen et al. (1954), Subbiah and Asija (1956), respectively. The dehydrogenase activity, which actually reflects the microbial activity in the soil, was determined by the method of Casida et al. (1964).

### Cluster and Principal Component Analysis

Cluster analysis categorizes multivariate data into subgroups through a wide range of methods. By shaping multivariate data into subsets, clustering can help reveal the attributes of existing structures or patterns. Hierarchical cluster analysis for agronomical and soil parameters was accomplished to construct a dendrogram based on the mean distance between treatments using the unweighted pair group method of arithmetic means (UPGMA). Principal components analysis (PCA) is the data reduction method relevant to quantitative data types. PCA transmutes multi-correlated variables into distinct sets of uncorrelated variables for further investigation, simplifying the complexity in high-dimensional data while preserving trends and patterns. Such new distinct sets of variables are linear amalgamations of original variables. This is based on the eigen values and mutually independent eigen vectors (principal components) arranged in the descending order of variance magnitude. Such components provide scatterplots of observations with the finest properties to examine the underlying correlation and variability. In order to estimate the relative effect of different treatments on agronomic and soil parameters, a PCA and cluster analysis were performed using the software PAST 4.03 (Hammer et al., 2001).

### Statistical Analysis

Before statistical analysis, data were normalized using the Shapiro-Wilk normality test. A statistical method was used to assess the suitability of treatments for different variables, such as agronomic parameters (height of the plant, number of tillers per hill, dry matter accumulation, panicle length, the number of panicles per hill, the number of grains per panicle, 1,000 grain weight, biological yield, grain Zn content, grain yield, straw yield, and harvest index), and soil parameters (pH, EC, organic carbon content, available nitrogen, phosphorus, potassium, and Zn). Data obtained from field trials of both regions (*Terai* and *Katchar*) were subjected to statistical analysis by one-way ANOVA using OPSTAT software packages^1^. The RBD with three replicates for each treatment was used for the experiments. Treatment means were compared by the Least Significant Difference (LSD) test with a probability of 5% (*p* < 0.05). The graphs were created using GraphPad Prism 5 software (GraphPad Software, San Diego, CA, United States).

## Results

### Zinc Solubilization Assay

**FIGURE 1:**
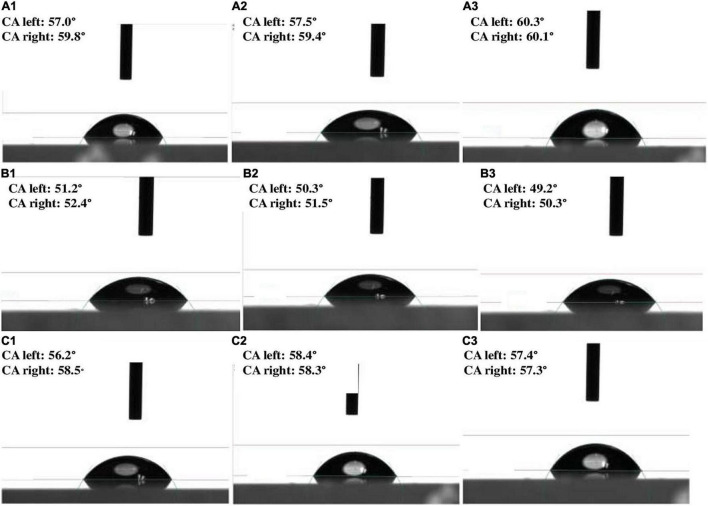
Contact angle images depicting various level of values of broth suspension: **(A1–A3)** (uninoculated control); **(B1–B3)** (BMRR126); and **(C1–C3)** (BMAR64) inoculated broth.

**FIGURE 2:**
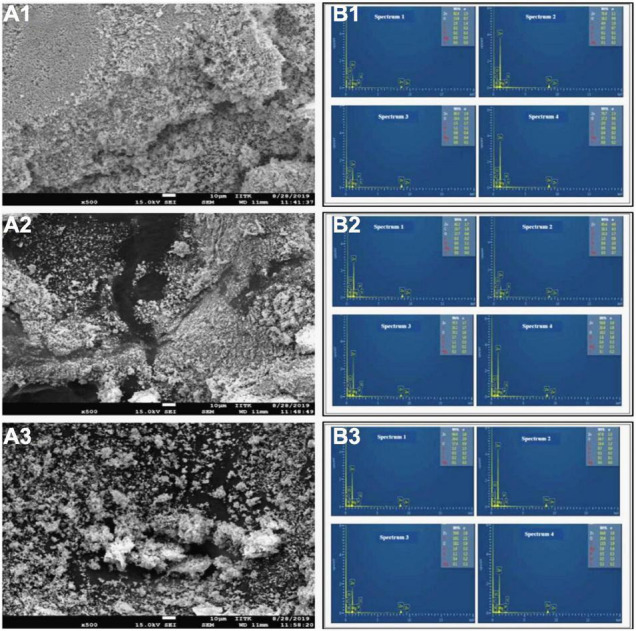
Scanning electron micoscopy (SEM) **(A1–A3)**, typical EDX spectrum **(B1–B3)** and element quantification analysis exhibit mobility of ZnO, showing element spectrum (Wt%) of Zn (zinc) and C (carbon) by isolates, BMRR126 **(A2,B2)** and BMAR64 **(A3,B3)** in Tris mineral salt medium culture and uninoculated Tris mineral salt medium depicts the insoluble zinc (Zn) source of ZnO **(A1,B1)**.

A total of 62 different bacterial isolates from rhizospheric soils of millet were examined for Zn solubilization potential. Finally, two bacterial isolates i.e., BMRR126 and BMAR64, were selected based on zinc solubilizing potential. The isolate, BMRR126, showed a larger Zn solubilizing halo zone (3.17 cm) compared to BMAR64 (2.40 cm) on Tris-mineral salts medium (amended with ZnO) (Table 3 and Supplementary Figure 1) but the highest solubilization efficiency (SE) was shown by BMAR64 (i.e., 1200.00%) (Table 3). Similarly, BMRR126 significantly augmented the Zn availability in the liquid broth amended with ZnO as compared to BMAR64 (Table 3). Evaluation of pH indicated a decrease in pH to a minimum of 4.38 from the initial pH of 7.2 after 1 week of incubation. The cell-free culture supernatant of BMRR126 exhibited the lowest pH value of 4.38 followed by BMAR64 with the value of 5.86. The lowering of the pH value of the inoculated broth medium shows an elevated acidity. The decreased value of the contact angle (50.23 for CA left and 51.40 for CA right) for the cell-free culture supernatant of BMRR126 compared to the uninoculated control shows the production of organic acids or other similar hydrophilic products in the cell-free suspension of the broth medium (Figure 1 and Table 3). The SEM images of ZnO amended Tris-mineral salt broth medium on the 10th day after incubation of both isolates exhibited better bio mobilization of Zn over non-inoculated control (Figure 2). The EDX analysis of four spectra also showed a decrease in the Zn percent (%) and an increase in the carbon content in the residues of the treated samples compared to the uninoculated control (Figure 2 and Supplementary Table 1).

**TABLE 3:** Zinc solubilizing potential of BMRR126 and BMAR64 in solid medium [qualitative Zn solubilization by measuring halo zone formation and solubilization efficiency (SE)] and liquid medium (quantitative Zn estimation, pH, and contact angle analysis).

ZSB isolates	Zinc solubilizing potential			pH of broth medium	Contact angle (CA) of broth medium	
	Qualitative assay		Quantitative assay			
	halo zone (cm)	Solubilization efficiency (%)	Solubilized zinc (mg/kg)		CA left	CA right
Uninoculated control	–	–	–	7.2	58.70 ± 0.85	58.57 ± 0.82
BMRR126	3.17 **±** 0.12	633.33	30.08 **±** 1.54	4.38 **±** 0.13	50.23 ± 0.38	51.40 ± 0.38
BMAR64	2.40 ± 0.26	1200.00	12.22 ± 0.61	5.86 ± 0.20	56.90 ± 0.58	59.23 ± 0.61

*Data are represented by mean value using three replicates.*

### Bacterial Characterization

**FIGURE 3:**
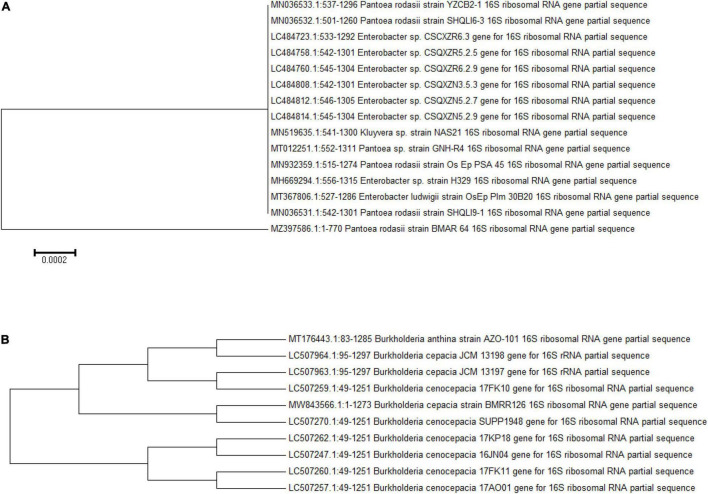
Phylogenetic tree of both ZSB isolates, BMAR64 **(A)** and BMRR126 **(B)**.

Both zinc-solubilizing bacterial isolates were attributed on basis of morphological and biochemical analysis (Supplementary Table 2). The 16S rDNA-based analysis revealed the confirmed identification of BMRR 126 as *Burkholderia cepacia* (accession number MW843566) and BMAR64 as *Pantoea rodasii* (accession number MZ397586) (Figure 3). The consequences of the BLAST search of the 16S rRNA gene sequences depicted the BMRR126 isolate as closely related to *Burkholderia cepacia*. On the other hand, BMAR64 showed the highest similarity with the *Pantoea rodasii* strain Os_Ep_PSA_45 with the representation of the closest homolog, *Pantoea* sp. Strain GNH-R4.

### Plant Growth Promoting Attributes

Both the bacterial isolates were further determined for different PGP traits. Isolates showed positive outcomes and gave a varying degree of results for all plant growth-promoting attributes (Table 4). The highest halo zone formation depicting the potential of siderophore production was recorded for BMRR126 (5.87 ± 1.03 cm) followed by BMAR64 (1.57 ± 0.15 cm). The maximum phosphate solubility was determined for BMRR126 (302.67 μg/ml) followed by BMAR64 (241.67 μg/ml) after 7 days of incubation. Isolates, BMRR126 and BMAR64, produced the IAA (auxin) but the greatest potential of IAA production was observed in BMAR64 at a level of 29.82 μg/ml, followed by the isolate, BMRR126 with a range of 23.45 μg/ml in tryptophan-amended broth. In addition, isolates also showed a positive result for EPS production. Both the isolates, namely BMRR126 and BMAR64, showed different levels of EPS production of 2.80 mg/ml and 2.18 mg/ml, respectively. Moreover, the qualitative test of ammonia production was found to be positive for both isolates (BMRR126 and BMAR64).

**TABLE 4:** Plant probiotic traits of potential zinc solubilizing bacterial isolates.

Isolates	Siderophore production	Phosphate solubilization	IAA production (μg/ml)	EPS Production (mg/ml)	Ammonia production
	Qualitative assay (cm)	Quantitative assay (μg/ml)			
BMRR126	5.87 ± 1.03	302.67 ± 6.03	23.45 ± 1.18	2.80 ± 0.10	+
BMAR64	1.57 ± 0.15	241.67 ± 4.73	29.82 ± 1.46	2.18 ± 0.18	+

*Data are represented by mean value using three replicates.*

*“ + ” indicates positive result.*

### Field Experiment and Biometric Observation

Field experiments were carried out at two subsequent locations to depict the prolific effects of ZSB strains (BMRR126 and BMAR64) and their consortium with and without Zn supplementation (ZnO). The ZSB isolates and their consortium have a positive effect on rice growth and influence plant height, number of tillers per hill, dry matter accumulation per hill, effective tillering, panicle length, number of grains per panicle, 1,000 grain weight, grain yield, and straw yield (Tables 5–7). The outcomes of the growth and yield parameters of paddy after treatments by ZSB and their consortium with and without an insoluble source of zinc (ZnO) exhibited varying levels of results. However, the least values were recorded for the uninoculated control treatment (T1). Moreover, the highest values for all the parameters were recorded for the *Katchar* region compared to the *Terai* region. Among the treatments, the maximum value of the plant height was recorded in the treatments containing ZSB inoculant, BMRR126 (137.39 cm), and consortium + ZnO (111.17 cm) for the *Katchar* region and *Terai* region, respectively. Similarly, the highest number of tillers hill^–1^ (13.27) was determined for the treatment T8 containing the bacterial strain, BMAR64, and ZnO supplementation for the *Terai* region as compared to the control (9.87). However, in the *Katchar* region, the highest number of tillers hill^–1^ (15.13) was calculated for the treatment T9 (consortium + ZnO) as compared to the control treatment (11.20).

**TABLE 5:** Effect on growth parameters (plant height, number of tillers, and dry matter accumulation) of rice at harvest stage under field experiment.

	Treatments details	Plant height (in cm)		No. of tillers hill^–1^		Dry matter (g) hill^–1^	
Labels		*Terai* region	*Katchar* region	*Terai* region	*Katchar* region	*Terai* region	*Katchar* region
T1	Control	100.66	126.85	9.87	11.20	45.60	46.26
T2	ZnSO_4_	102.73	130.85	11.93	12.33	46.21	49.61
T3	ZnO	101.19	128.75	10.60	11.87	46.00	46.22
T4	BMRR126	105.95	137.39	12.40	13.27	49.65	51.34
T5	BMAR64	103.93	132.49	12.27	13.67	47.04	50.72
T6	Consortium*	105.09	134.79	12.73	13.47	50.04	54.28
T7	BMRR126 + ZnO	108.13	134.61	13.13	13.93	50.71	55.87
T8	BMAR64 + ZnO	105.88	134.02	13.27	14.47	47.60	53.00
T9	Consortium* + ZnO	111.17	134.87	12.80	15.13	52.13	54.73
	SE(m)±	0.93	1.02	0.65	0.58	1.53	1.28
	C.D.	2.82	3.10	1.99	1.77	NS*	3.87
	C.V.	1.54	1.34	9.41	7.68	5.49	4.32

Each value is mean of three replicates. Data were analyzed statistically at the 5% (p < 0.05) level of significance. Consortium* contains bacterial strains, BMRR126 and BMAR64.

**TABLE 6:** Effect on growth parameters (effective tillers, panicle length, number of grains per panicle, and 1,000 grain weight) of rice at harvest stage under field experiment.

	Treatments details	Effective tillers		Panicle length (in cm)		No. of grain/panicle		1000 grain weight (g)	
Labels		*Terai* region	*Katchar* region	*Terai* region	*Katchar* region	*Terai* region	*Katchar* region	*Terai* region	*Katchar* region
T1	Control	9.20	10.53	28.92	30.25	130.87	142.83	16.6	18.38
T2	ZnSO_4_	11.07	11.40	30.25	31.47	147.07	167.03	17.84	20.24
T3	ZnO	9.67	11.33	29.32	30.67	137.20	155.17	16.81	19.32
T4	BMRR126	11.20	12.67	31.14	32.70	159.93	176.90	18.56	20.94
T5	BMAR64	10.80	12.73	30.04	32.22	154.40	171.33	18.14	19.13
T6	Consortium*	11.27	13.07	32.31	33.28	169.27	177.20	18.77	20.58
T7	BMRR126 + ZnO	12.33	13.40	32.44	34.32	172.40	185.33	19.85	22.37
T8	BMAR64 + ZnO	12.20	12.93	31.81	33.03	165.07	185.83	19.6	21.1
T9	Consortium* + ZnO	12.47	13.60	32.95	34.26	179.67	184.60	20.43	21.6
	SEM ±	0.64	0.70	0.49	0.62	1.98	2.77	0.36	0.46
	C.D.	1.95	NS*	1.48	1.87	5.99	8.39	1.11	1.39
	C.V.	10.06	9.77	2.37	3.30	2.18	2.79	3.43	3.91

Each value is mean of three replicates. Data were analyzed statistically at the 5% (p < 0.05) level of significance. Consortium* contains bacterial strains BMRR126 and BMAR64.

**TABLE 7:** Effect on yield parameters of rice at harvest stage under field experiment.

	Treatments details	Grain yield (q/ha)		Straw yield (q/ha)		Biological yield (q/ha)		Harvest index	
Labels		*Terai* region	*Katchar* region	*Terai* region	*Katchar* region	*Terai* region	*Katchar* region	*Terai* region	*Katchar* region
T1	Control	37.94	38.95	52.80	53.65	90.74	92.60	41.83	42.87
T2	ZnSO_4_	39.67	41.77	52.48	57.52	92.15	99.28	43.08	43.99
T3	ZnO	38.53	39.23	52.29	53.41	90.82	92.64	42.44	41.18
T4	BMRR126	40.77	41.96	57.69	59.15	98.45	101.11	41.41	43.96
T5	BMAR64	38.80	39.92	55.26	59.64	94.06	99.56	41.28	43.03
T6	Consortium*	41.27	43.13	58.58	70.93	99.85	114.07	41.36	43.44
T7	BMRR126 + ZnO	42.52	43.59	55.94	67.71	98.46	111.30	43.48	41.67
T8	BMAR64 + ZnO	39.95	41.89	53.08	64.26	93.04	106.15	43.44	42.41
T9	Consortium* + ZnO	41.57	43.56	57.54	62.28	99.11	105.84	41.96	43.41
	SEM ±	0.86	0.70	3.68	2.90	3.86	2.99	1.68	0.74
	C.D.	2.62	2.12	NS*	8.79	NS*	9.06	NS*	NS*
	C.V.	3.74	2.92	11.57	8.26	7.03	5.06	6.91	2.99

Each value is mean of three replicates. Data were analyzed statistically at the 5% (p < 0.05) level of significance. Consortium* contains bacterial strains, BMRR126 and BMAR64.

The treatment containing the consortium and the recommended dose of ZnO (T9) showed the highest values of dry matter accumulation hill^–1^ (52.13) for the *Terai* region, while for the *Katchar* region, the treatment of the bacterial strain, BMRR126, with ZnO supplementation (T7) depicted the highest dry matter accumulation hill^–1^ (55.87 gm). Moreover, the bacterial consortium with ZnO supplementation (T9) showed the highest values of the effective tillering for both regions, i.e., *Terai* region (12.47) and *Katchar* region (13.60), in comparison to the control treatment (9.20 for the *Terai* area and 10.53 for the *Katchar* area). The highest value of the panicle length (32.95 cm) was recorded for consortium + ZnO (T9) followed by treatments containing BMRR126 + ZnO (32.44 cm) and consortium (32.31) for the *Terai* region. In contrast, the maximum value of panicle length (34.32 cm) was observed for the treatment containing BMRR126 with ZnO supplementation (T7) followed by consortium + ZnO (T9) which showed the panicle length value as 34.26 cm. For the *Terai* region, T9 (consortium + ZnO) showed the highest digits for the number of grains per panicle (179.67) in comparison with other treatments, while the bacterial strain, BMAR64 with ZnO amendment (T8) had an utmost value for grains per panicle (185.83) for the *Katchar* region. The highest grain yield (q/ha) was measured for the BMRR126 + ZnO (T7) for both the regions, *Terai* (42.52 q ha) and *Katchar* (43.59 q/ha). But treatment containing the bacterial consortium with no ZnO supplement (T6) showed a maximum value for straw yield for the *Terai* region (58.58 q/ha) and *Katchar* region (70.93 q/ha) in comparison to control treatment (52.80 q/ha for the *Terai* region and 53.65 q/ha for the *Katchar* region). Likewise, the same treatment also showed the highest value of the biological yield for both regions (99.85 q/ha for the *Terai* region and 114.07 q/ha for the *Katchar* region) compared to the control treatment (90.74 q/ha for the *Terai* region and 92.60 q/ha for the *Katchar* region).

### Zinc Content Estimation in Grain

**FIGURE 4:**
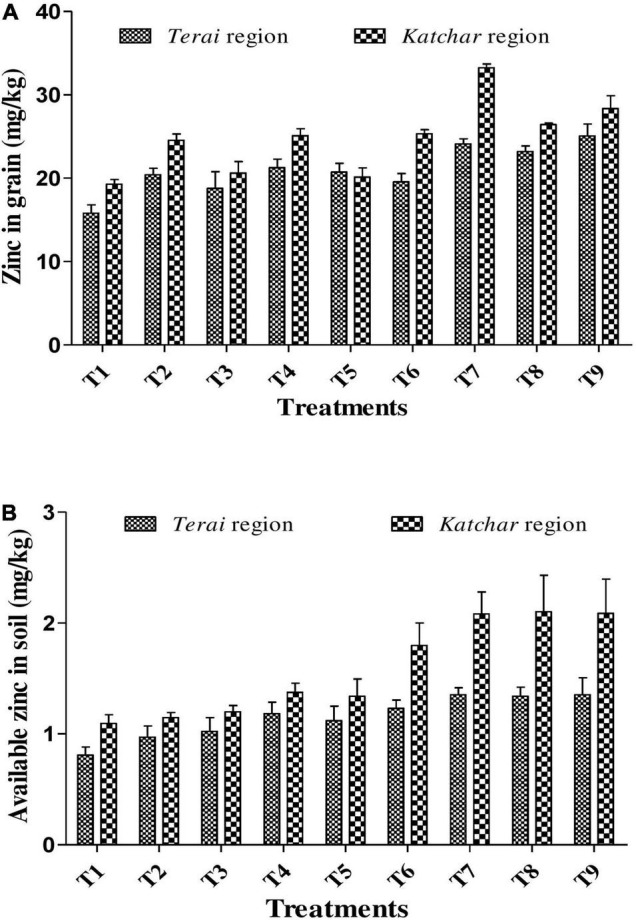
Concentration of Zn (mg/kg) in grain **(A)** and available Zn (mg/kg) in soils **(B)** for *Terai* and *Katchar* regions.

Inoculation of proficient ZSB strains and their consortium bearing massive PGP abilities not only augments plant growth but also increases the Zn micronutrient concentration in rice grains. Data about the Zn content in grains for all treatments from both trial sites are depicted in Figure 4A. The results from the *Terai* region revealed that the maximum Zn content (25.07 mg/kg) was observed for the treatment containing consortium with ZnO supplement (T9); there was almost 1.58-fold increment over the control (T1) which was 15.80 mg/kg. In the *Katchar* region, the highest Zn content (33.25 mg/kg) was observed for the BMRR126 + ZnO (T7), which showed a 1.72-fold increase compared to the control treatment (19.28 mg/kg). In both regions, all treatments showed significant results with a CD score of 2.83 and 2.92 for the *Terai* and *Katchar* regions, respectively (Supplementary Table 4). The results indicated that either single bacterial or consortium inoculation in combination with the ZnO application showed significant outcomes in providing Zn biofortification benefits *via* increasing the Zn concentration in edible parts of paddy.

### Chemical Properties of Soil

**FIGURE 5:**
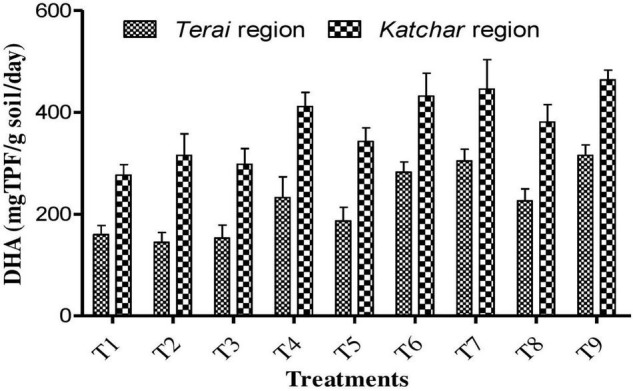
Dehydrogenase (DHA) activity of the soils in the *Terai* and *Katchar* regions.

Soil samples from both sites were collected from each treatment plot after the harvest of the crop for the determination of chemical properties, such as pH, EC, organic carbon, and NPK (Table 8). The soil pH value of the treated plots showed a lower value compared to the control. At both locations, the highest organic carbon was reported for the T7 (BMRR126 + ZnO), while the maximum P content was determined for T9 (consortium+ZnO). In addition, BMRR126 + ZnO (T7) for the *Terai* region and consortium + ZnO (T9) for the *Katchar* region showed the maximum value of N and K compared to other treatments. The data on the DTPA-extractable Zn in each treatment soil is shown in Figure 4B. The highest level of available Zn (1.35 mg/kg) is reported for BMRR126 + ZnO (T7) and consortium + ZnO (T9) for the *Terai* region. In contrast, BMAR64 + ZnO (T8) showed a relatively higher level of available Zn (2.10 mg/kg) for the *Katchar* region, followed by consortium + ZnO (T9) (2.09 mg/kg) and BMRR126 + ZnO (T7) (2.08 mg/kg). Furthermore, consortium + ZnO (T9) exhibited maximum DHA (dehydrogenase activity) for both *Terai* region (315.18 mgTPF/g soil/day) and *Katchar* region (463.41 mgTPF/g soil/day) (Figure 5).

**TABLE 8:** Chemical properties of the experimental soils of *Terai* and *Katchar* regions.

	Treatments details	pH		EC		Organic carbon (%)		Nitrogen (kg/ha)		Phosphorus (kg/ha)		Potassium (kg/ha)	
Labels		*Terai* region	*Katchar* region	*Terai* region	*Katchar* region	*Terai* region	*Katchar* region	*Terai* region	*Katchar* region	*Terai* region	*Katchar* region	*Terai* region	*Katchar* region
T1	Control	7.86	7.42	0.461	0.336	0.72	0.75	220.56	267.61	16.13	19.03	114.5	138.21
T2	ZnSO_4_	7.80	7.35	0.469	0.332	0.75	0.8	226.3	271.79	17.50	19.78	120.29	158.26
T3	ZnO	7.75	7.40	0.471	0.337	0.73	0.76	222.2	267.61	16.80	19.65	117.79	143.7
T4	BMRR126	7.62	7.30	0.335	0.281	0.79	1.12	245.21	288.51	20.01	21.81	139.81	183.27
T5	BMAR64	7.71	7.30	0.442	0.297	0.74	0.99	232.87	280.15	18.71	21.24	136.75	212.96
T6	Consortium*	7.56	7.18	0.331	0.269	0.77	0.81	226.22	284.33	19.08	21.2	144.59	150.52
T7	BMRR126 + ZnO	7.45	6.95	0.341	0.284	0.81	1.18	245.67	309.42	19.40	23.27	158.59	168.71
T8	BMAR64 + ZnO	7.64	7.23	0.348	0.295	0.77	1.1	231.37	305.24	19.28	22.03	142.13	201.67
T9	Consortium* + ZnO	7.48	6.91	0.334	0.271	0.79	0.86	238.34	317.78	20.06	23.44	155.57	226.35
	SE(m)	0.11	0.10	0.02	0.02	0.02	0.05	8.30	10.30	0.48	0.90	10.70	10.90
	C.D.	NS*	0.32	0.08	NS*	NS*	0.16	NS*	31.15	1.48	2.72	NS*	32.96
	C.V.	2.55	2.54	12.50	14.03	4.77	9.90	6.19	6.19	4.56	7.34	13.56	10.73

Each value is mean of three replicates. Data were analyzed statistically at the 5% (p < 0.05) level of significance. Consortium* contains bacterial strains, BMRR126 and BMAR64.

### Principal Component Analysis and Cluster Analysis

**FIGURE 6:**
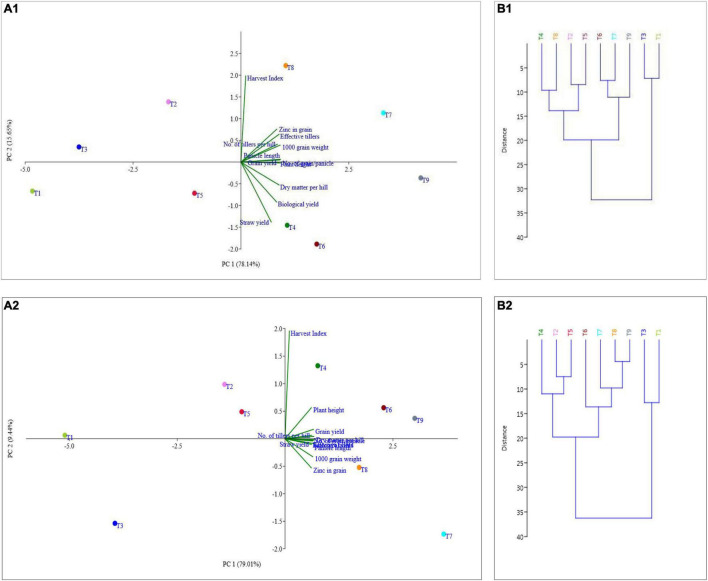
Relative performance prognosis of different treatments on agricultural parameters (plant height, number of tillers, effective tillers, dry matter, panicle length, number of grains/panicle, 1,000 grain weight, grain yield, straw yield, biological yield, harvest index, and Zn content in grain) through cluster (**B1** for the *Terai* region and **B2** for the *Katchar* region) and principle component analysis (PCA) (**A1** for the *Terai* region and **A2** for the *Katchar* region).

**FIGURE 7:**
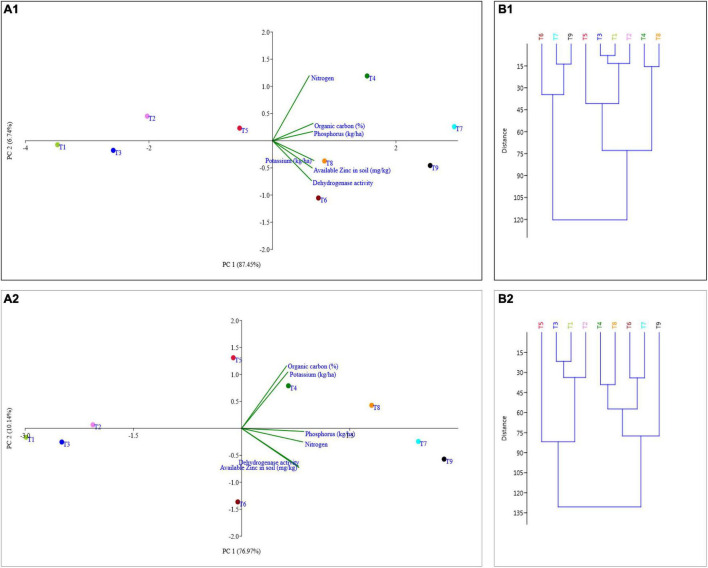
Representation of different treatments on soil parameters (organic carbon, nitrogen, phosphorus, potassium, available zinc, and dehydrogenase activity) through cluster (**B1** for the *Terai* region and **B2** for the *Katchar* region) and PCA (**A1** for the *Terai* region and **A2** for the *Katchar* region).

The principal component analysis allowed us to demonstrate the consequence of different treatment applications in a more cohesive mode between different variables under field experimentation in two different regions (Figures 6A1,A2, 7A1,A2). Two main components of PCAs for the agronomic parameters accounted for 93.79% of the experiment variability, i.e., PC1 for 78.14% and PC2 for 15.65%, for the *Terai* region (Figure 6A1). For the *Katchar* region, the PC1 and PC2 explained ∼88.45% of the total variability (PC1 for 79.01% and PC2 for 9.44%) (Figure 6A2). In addition, two main components of PCAs were determined for the soil-related parameters with 87.45% (PC1) and 6.74% (PC2) for the *Terai* region (Figure 7A1), while for the *Katchar* region, the values of PC1 and PC2 were determined as 76.97% and 10.14%, respectively (Figure 7A2). The concluding remarks of PCA, evidently indicate that two treatments, such as BMRR126 + ZnO (T7) and BMAR64 + ZnO (T8) in the *Terai* region, and three treatments, BMRR126 (T4), consortium (T6) and BMRR126 with ZnO supplementation (T7) in the *Katchar* region, displayed noteworthy improvement in agronomic parameters (Figure 6). While T4 and T7 in the *Terai* region and BMRR126 (T4) and BMAR64 with ZnO supplement (T8) in the *Katchar* region displayed noteworthy improvement in soil-related parameters. Outcomes of the cluster analysis are also similar to PCA (Figures 6B1,B2, 7B1,B2). The agronomical parameters, such as the number of grains per panicle for the *Terai* region and harvest index for the *Katchar* region had the maximum coefficient value for the PC1 (0.99121) and PC2 (0.97238), respectively (Table 9). The soil related parameters, such as phosphorus for the *Katchar* region and nitrogen for the *Terai* region had the maximum coefficient value for the PC1 (0.98927) and PC2 (0.48289), respectively (Table 10).

**TABLE 9:** Loading of coefficients of percentages of different variables of selected agronomical parameters for the first two principal components.

Variable	Principal components for the *Terai* region		Principal components for the *Katchar* region	
	PC 1	PC 2	PC 1	PC 2
Plant height	0.95826	0.023864	0.87717	0.27943
No. of tillers per hill	0.89968	0.19636	0.88837	0.029895
Dry matter per hill	0.94055	−0.25078	0.9884	−0.00893
Effective tillers	0.94076	0.3051	0.94802	−0.0143
Panicle length	0.9782	0.030622	0.98501	−0.04504
No. of grain/panicle	0.99121	−0.00974	0.96147	0.02044
1000 grain weight	0.96764	0.18626	0.92082	−0.16241
Grain yield	0.93552	−0.00529	0.94412	0.084063
Straw yield	0.74778	−0.65762	0.8781	−0.05142
Biological yield	0.87994	−0.43816	0.92305	−0.02079
Harvest Index	0.11875	0.94506	0.14513	0.97238
Zinc in grain	0.88373	0.36075	0.87716	−0.26355
% of Variance	78.14	15.65	79.01	9.44
Eigen value	9.37	1.87	9.48	1.13

**TABLE 10:** Loading of coefficients of percentages of different variables of selected soil-related parameters for the first two principal components.

Variable	Principal components for the *Terai* region		Principal components for the *Katchar* region	
	PC 1	PC 2	PC 1	PC 2
Organic carbon (%)	0.95681	0.12827	0.71379	0.48229
Nitrogen	0.86659	0.48289	0.97315	−0.10445
Phosphorus (kg/ha)	0.94897	0.068391	0.98927	−0.02397
Potassium (kg/ha)	0.97747	−0.1469	0.73391	0.43436
Available Zinc in soil (mg/kg)	0.93825	−0.20242	0.90994	−0.30181
Dehydrogenase activity	0.91894	−0.29664	0.90377	−0.29104
% of Variance	87.45	6.74	76.97	10.14
Eigen value	5.24	0.40	4.61	0.60

## Discussion

“Microbial assisted biofortification” is a novel concept in the field of agricultural microbiology for nutrifying crop edibles with essential micronutrients (Zn, Fe, and Se). Zn is an indispensable element required for the maintenance of vital activities in humans, animals, and plants. The lack of this micronutrient triggers the onset of Zn malnutrition-linked ailments. In many regions of northern India, rice cultivation is being more popular among middle-income farmers as it provides the staple food for a large section of the South Asian population. India, as the second-largest rice producer, produced 112.76 million tons of rice in 2017-2018 (Sanjeeva Rao et al., 2020). At present, rice cultivation in North India is gaining popularity as these regions have all the necessary facilities, mainly irrigation systems, for rice cultivation. In fact, rice grain is the main source of calorie intake in many developing countries, but the Zn content in rice is insufficient to achieve the appropriate Zn content for human consumption (Zaman et al., 2018; Chávez-Dulanto et al., 2021). Hence, the present study reveals the incredibility of potential ZSB in improving general crop growth parameters that show the benefit of Zn biofortification of rice grain at two different state-wise regions of northern India. In the current investigation, BMRR126 and BMAR64 were recognized as competent ZSB from the rhizosphere of the barnyard millet. Rhizospheric soil is the main hub for soil-dwelling microorganisms, where a diverse range of microorganisms possibly solubilize the insoluble form of Zn and likewise augment the crop yield (Khan et al., 2019; Upadhayay et al., 2021, 2022).

### Zinc Solubilization Potential

In the current study, BMRR126 and BMAR64 were identified as the most effective Zn solubilizers. Though the strain BMRR126 showed a maximum area of halo zone illustrating Zn solubilization (3.17 cm), strain BMAR64 exhibited higher Zn SE (1200%) on ZnO supplemented medium (Table 3). A previous study carried out by Shaikh and Saraf (2017) also observed the solubilization of Zn on ZnO-supplemented medium, where *Exiguobacterium aurantiacum* (MS-ZT10) produced a clear zone of 31 mm in ZnO-containing medium. The possibility of Zn solubilization could be due to the stronger adhesion to insoluble metal residues (Saravanan et al., 2004). Further confirmation of Zn solubilization was evaluated in a liquid broth medium amended with ZnO as an insoluble 0.1% of Zn source. The results revealed that the potentiality of the strain, BMRR126 exerted promising Zn solubilization in comparison to strain BMAR64. These similar patterns of outcomes were also depicted by Ramesh et al. (2014), where two *Bacillus* strains (MDSR7 and MDSR14) efficiently solubilized the ZnO as an insoluble Zn compound. The decline in the pH of the inoculated liquid broth, as opposed to uninoculated broth (pH 7.2) (Table 3), clearly indicated the secretion of organic acids in the broth medium. The secretion of gluconic acid by ZSB was considered a prime feature for Zn solubilization (Fasim et al., 2002; Mumtaz et al., 2017; Kushwaha et al., 2020; Prathap et al., 2022; Upadhayay et al., 2022). Besides, other organic acids including oxalic, tartaric, formic, (Li et al., 2010) lactic acid, malonic acid, citric acid (Vidyashree et al., 2018a,b), and acetic acids (Mumtaz et al., 2019) which have been detected in the broth medium inoculated with ZSB, might have a probable role in the solubilization of insoluble Zn. The higher availability of elemental Zn is directly related to the acidic pH of a liquid medium, which explicates the mechanism of bacterial-assisted Zn solubilization (Desai et al., 2012). Pieces of literature have also suggested the auxiliary role of siderophore and protons produced by bacteria in Zn solubilization (Kamran et al., 2017; Hussain et al., 2018; Upadhayay et al., 2018, 2019, 2022). The lowest contact angle value which was detected for the inoculated liquid medium further illustrates the higher hydrophilicity of the liquid broth than that of the untreated control broth (Figure 1 and Table 3). It was the first report based on contact angle analysis of a tiny drop of Zn-supplemented broth indicating the presence of more hydrophilic compounds, such as organic acids, which are essential for Zn solubilization. Both strains were identified as *Burkholderia cepacia* and *Pantoea rodasii* based on biochemical tests and molecular characterization, respectively (Figure 3). However, an inadequate number of studies are available on Zn solubilization by *Burkholderia* and *Pantoea* sp.

### Field Emission-Scanning Electron Microscope/Energy Dispersive X-ray Analysis for Zinc Solubilization

The SEM-based analysis shows an effective mobilization of ZnO by both Zn solubilizing bacterial isolates compared to the control (Figure 2). The untreated control exhibits amorphous and dense residues of insoluble substances under SEM (Figure 2A1), while bacterial-treated samples showed lesser opaque “mineral residues” (Figures 2A2,A3). Our results were according to the findings of Gandhi and Muralidharan (2016), where the bacterium *Acinetobacter*, isolated from rice rhizosphere, efficiently mobilized ZnO observed under SEM. Elemental analysis of residues by EDX also depicted different spectra of elements, particularly, Zn and carbon (Figures 2B1–B3 and Supplementary Table 1). The values of the reduced percentage of Zn from four spectra of all samples indicate that both ZSB strains have effectively solubilized Zn compared to the control. The increased carbon content in samples inoculated with bacteria in all spectra indicated that organic compounds produced by bacteria may be aggregated with mineral residues.

### Plant Growth Promoting Behaviors

Both bacterial strains contained some selected plant probiotic traits and showed different results under *in vitro* studies (Table 4). The characteristic of siderophore production shows the iron-solubilizing behavior of both isolates and enhances the value of such isolates in the form of bio-inoculants to produce Fe-fortified crops. The trait of phosphate solubilization show an imperative role of the bacterial inoculant for crop vitalization (Singh et al., 2013). Both isolates also showed varying levels of P solubilization when measured quantitatively. The highest value (302.67 μg/ml) of phosphate solubilization by isolate BMRR126 further illustrates the remarkable potential of ZSB as a “P-solubilizer.” The earlier finding of Verma et al. (2020) reported “*Rhizobium radiobacter LB2*” for the phosphate solubilizing property besides being an efficient Zn solubilizer. These results are also in agreement with Mumtaz et al. (2017), Bhatt and Maheshwari (2020) who reported the phosphate solubilization potential of ZSB isolated from different sources. Isolates were also tested for IAA production and the results revealed that both ZSB strains contain the IAA production efficacy in the presence of L-tryptophan in the medium. There was considerable variation in IAA production between the ZSB isolates, ranging from 23.45 to 29.82 μg/ml in the L-tryptophan supplemented broth. In the past, a variation in the IAA production has been reported for ZSB isolates from the rhizosphere of *Chickpea and rice*, as reported by Gandhi and Muralidharan (2016), Zaheer et al. (2019), respectively. It is a well-established fact that an ample level of IAA increases plant growth and assists in the development of plant roots (Xie et al., 1996). In the present study, both ZSB isolates were evaluated for the production of EPS (exopolysaccharides), which provides help in effective root colonization. The plant facilitates the colonization of microbes by producing exudates that serve as a nutrient for bacteria in the rhizosphere (Bhattacharjee et al., 2012). In previous studies, the ZSB isolates, which demonstrate the ability to produce EPS, were used in the Zn enrichment of crops (Kamran et al., 2017; Mumtaz et al., 2017). The bacterial isolates also exhibited plant growth elevating features through the production of ammonia. This characteristic is also attributed to the plant growth-stimulating effect by plant-growth-promoting bacteria (Hayat et al., 2010; Backer et al., 2018).

### Growth Parameters and Yield-Related Attributes

Outcomes of the present study revealed the remarkable influence of ZSB and their consortium along with ZnO supplement on numerous vegetative growth parameters (plant height, number of tillers per hill, and dry matter accumulation) and yield-related attributes (effective tillering per hill, panicle length, number of grains/panicle, 1,000 grain weight, grain yield, and straw yield) over uninoculated control under field study carried out at two different regions viz *Terai* and *Katchar* regions. Bacteria with diverse plant growth-promoting properties show high potential to improve plant height, dry weight, and the number of tillers, especially when applied in combination or with Zn supplementation (Vaid et al., 2014; Doni et al., 2022; Prathap et al., 2022). Maximum plant height in response to the bacterial consortium with Zn for the *Terai* region and a single bacterial inoculation (BMRR126) for the *Katchar* region indicated a microbial-assisted improvement in plant height (Table 5). The variation in plant height at both locations must be due to certain factors including soil type and climatic conditions. The ZSB including *Acinetobacter sp, Burkholderia cenocepacia*, and *Serratia* sp exhibited remarkable improvement in rice plant height (Idayu Othman et al., 2017; Nepomuceno et al., 2020). The effect on the number of tillers was higher when ZSB were co-inoculated with ZnO for both regions (Table 5). Similar results were also reported by Shakeel et al. (2015) in Basmati rice cultivars in response to co-inoculation of ZSB with Zn supplementation. Calculating effective tillering is an important feature to express grain yield and productivity in response to a given treatment. All bacterial-inoculated treatments expressed significant numbers of effective tillers (Table 6). Previous studies demonstrated the role of *Burkholderia* and *Acinetobacter* in considerable increment in the number of effective tillers of paddy (Trân Van et al., 2000; Vaid et al., 2014). The field investigation also revealed maximum values of 1,000 grain weight in response to bacteria or their consortium with ZnO supplementation (Table 6). Similar findings by Gandhi and Muralidharan (2016) also illustrated the effect of *Acinetobacter* with Zn supplement on increasing the 1,000 grain weight of rice. The effect of the bacterial strain, BMRR126 in combination with ZnO (T7) on the grain yield in a significant manner at different locations shows the better effectiveness of the bacterial isolate with Zn fertilizers (Table 7). Multitude studies decoded the enhanced grain yield in response to ZSB inoculants (Tariq et al., 2007; Vaid et al., 2014; Hussain et al., 2018; Kushwaha et al., 2020; Prathap et al., 2022). The study of Shakeel et al. (2015) illustrated the prolific effect of *Bacillus* sp on the rice plant in terms of increasing the grain yield. Bacteria play an auxiliary factor in the assimilation of nutrients from the soil, thereby increasing the grain yield and also crop biomass (Tariq et al., 2007; Suseelendra, 2012; Batool et al., 2021). The consortium of BMRR126 and BMAR64 in the paddy field gave the maximum biological yield (114.07 q/ha) for the *Katchar* region (Table 7). The result is similar to the result from the study of Shakeel et al., 2015, where the co-inoculation of ZSB and Zn showed an increased pattern of biological yield. In this study, results indicated that inoculation of ZSB resulted in an increase in straw yield (Table 7) which is supported by the results of Gandhi and Muralidharan (2016). However, the ZSB application along with ZnO showed an overall positive effect on the growth and yield of rice crops compared to a single Zn treatment containing ZnSO_4_ and ZnO. It might be due to the steady and slow release of Zn from ZnO along with the proficient act of ZSB (Zeb et al., 2018). Somehow, the similar patterns of studies of Vaid et al. (2014), Gandhi and Muralidharan (2016), also unveiled an apparent role of ZSB in terms of improving the growth-related parameters of rice plants.

### Zinc Content in Grain

The considerable augmentation in the Zn concentration of paddy variety, *Pusa Basmati*-1, was observed in the current study due to the effect of ZSB containing massive plant probiotic traits. Thus, the maximum Zn acquirement in the grain was determined for the treatment containing consortium and recommended dose of ZnO (T9) (for the *Terai* region) and BMRR126 + ZnO (T7) (for the *Katchar* region) (Figure 4A), where the application of consortium (BMRR126 and BMAR64) along with ZnO at *Terai* region and single ZSB bioinoculant BMRR126 at *Katchar* region confirm their ability of Zn solubilization. The results clearly showed that treatment with ZSB inoculants along with ZnO additives showed an improved pattern of Zn consignation in rice grains at both locations. The study by Shakeel et al. (2015), Gandhi and Muralidharan (2016) shows similar result patterns in which the ZSB shows a positive influence on the uptake of the elemental zinc in the rice grains. The key benefit of using ZSB is its dual action, firstly providing the biofortification benefits of rice to address the problem of zinc malnutrition, and secondly as a potential biofertilizer contributing to sustainable agriculture. The ZSB strains possess the plant probiotic properties to augment plant growth and improved nutrient acquisition for maintaining plant health (Upadhayay et al., 2022). The improved Zn concentration of grain is certainly influenced by ZSB inoculants, suggesting that the use of such microorganisms may offer green technological approaches for the biofortification of plants (Khan et al., 2019; Kushwaha et al., 2021). The increase in plant growth by ZSB is due to its imperative traits of root colonization (Idayu Othman et al., 2017). In our study, the augmented Zn levels in rice grain with the ZSB inoculants, BMRR126 and BMAR64, were viewed as an auxiliary tactic to curtail the Zn deficiency in cultivated rice at two consecutive locations (*Terai* region and *Katchar* region). The increased Zn content in the edible part of the plant may be due to efficient ZSB colonization, where organic acids secreted by bacteria lower the pH of the rhizospheric soil and create a favorable environment for Zn solubilization (Mumtaz et al., 2017; Hussain et al., 2018; Upadhayay et al., 2022). The finding of Zn content was consistent with previous studies using a ZSB microbial strain, such as *Bacillus* sp., enhanced Zn translocation (22–49%) in *Basmati* rice (*Basmati*-385) (Shakeel et al., 2015). Furthermore, Wang et al. (2014) deciphered that “*Enterobacter* sp.” and “*Sphingomonas* sp.” increased Zn content by 11.2% and 13.7% in polished rice, respectively.

### Available Zinc in Soil

The increased level of DTPA-extractable Zn content in soils due to bacterial treatments with ZnO additives has rectified the issue of more prevalence of an inaccessible form of Zn in soil (Figure 4B). It is well studied that ZSB solubilizes the complex type of Zn in the soil and makes it accessible to the plant system (Ramesh et al., 2014; Kushwaha et al., 2020; Upadhayay et al., 2021). The increased level of available Zn in soil for the treatment containing bacteria and Zn supplementation might be due to the organic acid production ability of inoculated bacteria which might slowly release zinc from ZnO and other insoluble Zn forms. Plenty of previous studies documented the various organic acid producing microorganisms assisting in lowering the rhizospheric soil pH (Ramesh et al., 2014; Shakeel et al., 2015; Krithika and Balachandar, 2016; Kushwaha et al., 2020). Moreover, Saravanan et al. (2004), Sarathambal et al. (2010) deciphered the decline of soil pH due to the inoculation of *G. diazotrophicus, Bacillus*, and *Pseudomonas* for sufficient Zn accessibility to plant. Improved soil Zn content after bacterial inoculation can be a good indicator of microbial activity and its fruitful contribution to enhancing Zn uptake by plants.

### Soil Parameters

Indeed, under field conditions, multiple external determinants come to the front and can reduce the capacity of soil bacteria to generate fertile impacts on plant growth. In field conditions, the bacterial inoculant must show competitiveness and withstand certain environmental stresses while retaining specific prolific traits. The microbiological activity in the soil should be measured using certain soil parameters, such as the available proportion of nitrogen, phosphorus, potassium, the content of organic carbon, and the determination of dehydrogenase activities. All treatments containing bacterial inoculants showed a pattern of reduced soil pH compared to no bacterial treatment (Table 8). The reduced soil pH may be due to the secretion of organic acids by ZSB (Fasim et al., 2002; Upadhayay et al., 2018, 2019; Kushwaha et al., 2020), and this phenomenon ultimately improves the availability of Zn in the soil (Saravanan et al., 2004; Upadhayay et al., 2022). The treatment containing BMRR126 with ZnO showed the highest organic carbon content (1.18%) in the *Katchar* region (Table 8). Since chemical-based fertilizers cannot increase soil organic matter, microbial inoculants improve soil organic carbon by promoting the mineralization of soil organic matter. Furthermore, Shahdi Kumleh (2020) illustrated the elevated soil organic carbon levels (1.79%) in response to bacterial inoculation in a clover rice cultivation system. Overall, a bacterial consortium with Zn supplementation, i.e., T9, showed maximum values of available nitrogen and phosphorus in the soil of both these regions (Table 8). BMRR126 and consortium in combination with ZnO depicted the highest level of available potassium in the soil of *Terai* region (158.59 kg/ha) and *Katchar* region (226.35 kg/ha), respectively (Table 8). The increased dehydrogenase activity in all bacterial inoculated treatments reflects the splendid microbial activity in the soil system. A recent report by Kumawat et al. (2022) examined the nutrients and enzymatic activities in the soil and extrapolated the increased dehydrogenase activity (32.66 μg triphenylformazan g^–1^ h^–1^) under the treatment of *Bradyrhizobium* sp., LSBR-3 and *Pseudomonas oryzihabitans* L compared to the control treatment. Microorganisms that produce organic acids facilitate the solubilization of bound phosphorus and potassium in the soil, thereby increasing the levels of these nutrients in the soil (Alori et al., 2017; Tian et al., 2021). Our study is justifiable with the previous studies in which bacterial treatments improved soil health by showing significant levels of organic carbon, nitrogen, phosphorus, and potassium (Adak et al., 2014; Reyes-Castillo et al., 2019).

Finally, we rationalize our study and present its uniqueness from other studies based on the following aspects:(i) EDX spectrum and contact angle-based analysis were new techniques used to assert the Zn-solubilization potential of bacteria isolated from rhizospheric soil of underutilized crop, i.e., barnyard millet; (ii) decoding the effects of ZSB with low-cost ZnO source input on multiple agronomic and soil parameters at two different sites.

Overall, our study showed that the agro parameters of rice crops were improved by the bacterial treatment together with the ZnO application in the soil. Similarly, bacterial inoculant in combination with soil zinc (ZnO) application increased the Zn content of grain (Figure 4A) in both agro-climatically different regions and proved to be a sustainable strategy for bacterial assisted grain biofortification. However, the application of ZnSO_4_ is common as zinc fertilizer and is an important part of agronomic Zn biofortification (Xu et al., 2021). The cost of ZnO is relatively lower compared to Zn sulfate; hence the present study recommends using ZnO as a Zn supplement with ZSB to solve the purpose of Zn malnutrition in a very economically feasible way.

## Conclusion

Zinc has a pivotal role in human health and also in crop production. The intensive cropping patterns, the rampant usage of chemical fertilizers, and a dearth of available soil zinc (Zn) results in inadequate consignation of the proper level of Zn in rice edibles and hence set forth the phenomenon of Zn malnutrition. Therefore, microbial-mediated biofortification can be a prolific tactic to counteract the issue of Zn malnutrition, as soil-dwelling ZSB acts as auxiliary natural factors to prop up plants for elevated Zn uptake from the soil system. The study was conducted to illustrate the ZnO solubilization ability of the two rhizospheric strains from the Himalayan underutilized crop, “barnyard millet.” The FE-SEM-EDX-based analysis revealed a notable Zn solubilization behavior by the mobility of zinc from the ZnO residue for both isolates, i.e., BMRR126 and BMAR64. The isolates were prominent for various plant probiotic traits, such as P solubilization, production of siderophores, IAA, EPS, etc. (Table 4). Further, both ZSB strains were determined under the field conditions at two different sites (*Terai* region and *Katchar* region) and demonstrated their auspicious effects in terms of showing maximum growth-yield related characteristics of paddy. The bacterial consortium for the *Terai* region and a bacterial inoculant (BMRR126) for the *Katchar* region with ZnO supplementation exhibited a 1.58- and 1.72-fold increase in Zn levels of the rice grain, respectively, compared to the control (Figure 4A). The improved soil quality was also evident with all treatments containing bacterial inoculants (Table 8). By carefully monitoring all parameters, this study proposes the use of ZSB inoculants with an economically feasible Zn source, specifically ZnO, to achieve the benefits of the overall plant productivity and the benefits of Zn biofortification. The outcomes of the current investigation motivate the authors to further explore the bacterial potentiality under various agro-climatic conditions to assess the agronomic and zinc biofortification benefits of multiple staple food crops.

## Data Availability Statement

The datasets presented in this study can be found in online repositories. The names of the repository/repositories andw accession number(s) can be found in the article/Supplementary Material.

## Author Contributions

VU wrote the original draft preparation. AVS did the conceptualization, performed the methodology and inputs for framing of manuscript, and supervised the data. AK and JS edited and reviewed the manuscript. NP validated the data. AR provided technical assistance in SEM-EDX and contact angle analysis. All authors contributed to the article and approved the submitted version.

## Conflict of Interest

The authors declare that the research was conducted in the absence of any commercial or financial relationships that could be construed as a potential conflict of interest.

## Publisher’s Note

All claims expressed in this article are solely those of the authors and do not necessarily represent those of their affiliated organizations, or those of the publisher, the editors and the reviewers. Any product that may be evaluated in this article, or claim that may be made by its manufacturer, is not guaranteed or endorsed by the publisher.

## Footnotes

1
http://14.139.232.166/opstat/
