# Supplementary material for: FE-SEM/EDX Based Zinc Mobilization Analysis of Burkholderia cepacia and Pantoea rodasii and Their Functional Annotation in Crop Productivity, Soil Quality, and Zinc Biofortification of Paddy
Source: Front Microbiol. 2022 May 6;13:852192. doi: 10.3389/fmicb.2022.852192 (PMC9120762; doi:10.3389/fmicb.2022.852192)
Supplement: Supplementary file 1 [file Data_Sheet_1.docx]

**Supplementary data:**

**Supplementary Table 1:** Element spectrum (Wt % of ‘Zn’ and ‘C’) of mineral residues from 10 days old Tris mineral salt medium amended under response of isolates BMRR126 and BMAR64

| **Bacterial isolates** | **Element**  **(Wt%)** | **Spectrum 1** | **Spectrum 2** | **Spectrum 3** | **Spectrum 4** |
| --- | --- | --- | --- | --- | --- |
| **Control** | **Zn** | 82.8 | 76 | 80.3 | 79.7 |
|  | **C** | 2.9 | 4.9 | 1.5 | 2.0 |
| **BMRR126** | **Zn** | 62.2 | 65.4 | 55.5 | 58.8 |
|  | **C** | 19.7 | 18.3 | 20.2 | 20.4 |
| **BMAR64** | **Zn** | 60.6 | 67.8 | 59.6 | 64.0 |
|  | **C** | 20.0 | 10.4 | 19.1 | 13.5 |

**Supplementary Table 2:** Biochemical characteristics of selected zinc solubilizing bacterial isolates

| **Morphology and biochemical**  **characteristics** | **ZSB isolates** | |
| --- | --- | --- |
|  | **BMRR126** | **BMAR64** |
| Gram’s stain | - | - |
| Cell shape | Rod | Rod |
| Colony pigmentation | Creamish-white | Light-yellowish |
| Indole test | **-** | **-** |
| Voges Proskauer | **-** | **+** |
| Methyl red | **-** | **-** |
| Citrate utilization | **+** | **+** |
| Catalase test | **-** | **-** |
| Gelatinase test | **-** | **-** |
| Caseinase test | **+** | **-** |
| Cellulase test | **-** | **-** |
| Amylase test | **-** | **-** |
| Urease test | **+** | **-** |

^‘+’ indicates positive result, ‘-’ indicates negative result^

**Supplementary Table 3**: Physiochemical properties of field soils of *Terai* region and *Katchar* region

| **Soil characteristics** | ***Terai* region** | ***Katchar* region** |
| --- | --- | --- |
| pH | 7.85 | 7.50 |
| EC | 0.4 | 0.3 |
| Organic carbon (%) | 0.68 | 0.73 |
| N (kg/ha) | 211 | 220.52 |
| P(kg/ha) | 15.7 | 17.04 |
| K(kg/ha) | 106 | 142.48 |
| Available Zn (mg/kg) | 0.72 | 1.02 |

|  | **Zn content in grain** | | **Available Zn content in soil** | | **Dehydrogenase activity of soil** | |
| --- | --- | --- | --- | --- | --- | --- |
|  | ***Terai region*** | ***Katchar region*** | ***Terai region*** | ***Katchar region*** | ***Terai region*** | ***Katchar region*** |
| **SE(m)** | 0.94 | 0.96 | 0.11 | 0.19 | 21.73 | 36.88 |
| **C.D.** | 2.83 | 2.92 | 0.32 | 0.58 | 65.71 | 111.52 |

**Supplementary Table 4:** Standard errors and CD of graphically represented results of grain Zn content, available zinc and dehydrogenase activity of soils in the *Terai* region and the *Katchar* region


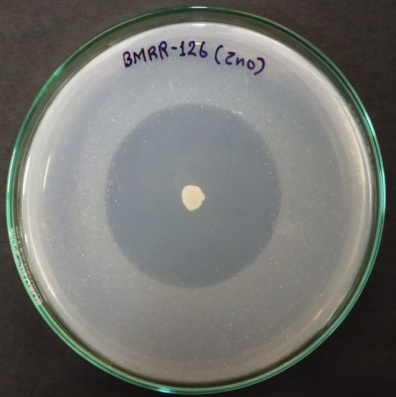

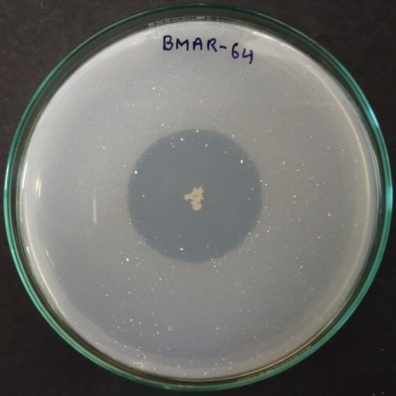


**b**

**a**

**Supplementary figure 1:** Figure depicting the zinc oxide solubilization in Tris minimal medium by bacterial isolates (a) BMRR126 (b) BMAR64
